# Promising Vaccine Formulations for Emerging Infectious Diseases

**DOI:** 10.3390/ijms26104893

**Published:** 2025-05-20

**Authors:** Pil-Gu Park, Seok-Yong Lee, Hyewon Youn, Kee-Jong Hong

**Affiliations:** 1 Department of Life Science, Gachon University, Seongnam 13120, Republic of Korea; ppkmtg81@gachon.ac.kr; 2 Department of Nuclear Medicine, Cancer Imaging Center, Seoul National University Hospital, Seoul 03080, Republic of Korea; iamlsy25451@snu.ac.kr; 3 Cancer Research Institute, Seoul National University College of Medicine, Seoul 03080, Republic of Korea; 4 Department of Health Sciences and Technology, Gachon Advanced Institute for Health Sciences & Technology (GAIHST), Gachon University, Incheon 21999, Republic of Korea; 5 Department of Microbiology, Gachon University College of Medicine, Incheon 21936, Republic of Korea; 6 Lee Gil Ya Cancer and Diabetes Institute, Gachon University, Incheon 21999, Republic of Korea; 7 Korea mRNA Vaccine Initiative, Gachon University, Seongnam 13120, Republic of Korea

**Keywords:** emerging infectious disease (EID), vaccine formulation, mRNA vaccine

## Abstract

Emerging infectious diseases (EIDs) are one of the greatest threats to human health today, thus requiring an urgent response. Vaccines are one of the most effective means of preventing the spread of infectious diseases, and their usefulness in responding to EIDs has been clearly proven through the process of overcoming the global COVID-19 pandemic. As the characteristics of various vaccine formulations differ, it is necessary to apply the most appropriate one according to the EID response strategy. In this review, we first consider which vaccine formulation is the most suitable for EID vaccines by comparing the pros and cons of different vaccine formulations, and then we discuss the utility of mRNA vaccine formulations, which are considered the most promising for EID vaccines.

## 1. Introduction

Today, various new infectious diseases are emerging and threatening human health. This includes a wide array of ailments, such as coronavirus 2019 (COVID-19), which recently caused a global pandemic [1]; influenza, which causes annually recurring epidemics [2]; deadly viral hemorrhagic fevers, such as Ebola, Marburg, Crimean–Congo, and Lassa [3,4,5]; gastrointestinal diseases triggered by enterotoxigenic *Escherichia coli* (ETEC) and Shigella [6]; fatal encephalitis induced by the Nipah virus and Japanese encephalitis virus (JEV) [7,8]. Additionally, diseases such as cholera, malaria, and tuberculosis, which continue to claim numerous lives in low- and middle-income countries, are also part of this list [9,10,11]. Chronic infections with hepatitis B and C viruses, human papillomavirus (HPV), and Epstein–Barr virus (EBV) can cause cancer [12,13,14,15]. Specifically, the number of emerging infectious diseases (EIDs) is increasing, posing a global health security risk, as traditional treatment or prevention methods prove insufficient. The global COVID-19 pandemic in 2019 resulted in severe human, economic, and social losses, highlighting the need to understand the potential implications of the emergence of EIDs. Consequently, these diseases pose a significant threat to human health, necessitating the development of strategies to combat them.

Humanity is actively combating infectious diseases through treatment with therapeutic agents and preventive measures such as vaccines. Therapeutic agents can be effective against infectious diseases with mild or progressive symptoms; however, they may not work for acute infectious diseases where symptoms rapidly worsen or for newly emerged drug-resistant pathogens [16]. In particular, an immediate response is essential to suppress the spread when an EID occurs; however, there is inevitably insufficient time to develop treatments tailored to the characteristics of each newly emerging causative pathogen.

The key to an effective response strategy for EIDs lies in preemptive prevention rather than post-treatment. Unlike therapeutic agents that are effective only for individuals already infected, vaccines can form protective immunity before the spread of an EID, blocking the spread of the pathogen itself and protecting high-risk groups such as the elderly, the immunocompromised, and infants before infection. The appropriate application of vaccines can significantly reduce the spread of infection through herd immunity and end pandemics early. While the role of therapeutics is, of course, important in responding to EIDs, vaccines are the most effective first-line defense measure to prevent the rapid spread of novel infectious diseases and reduce social damage. Therefore, in order to respond to EIDs, it is crucial to develop a prophylactic vaccine based on the most expeditious platform that can preemptively suppress infections caused by pathogens.

This review presents trends in vaccine development research to address EIDs that currently threaten global health security. By examining the pros and cons of various formulations that can be applied to EID preventive vaccines and by proposing the most promising vaccine formulations, we aim to provide insights for effective EID responses in the future.

## 2. Current Trends in EIDs

EIDs typically occur when existing pathogens acquire new characteristics through mutation [17]. Characteristic changes leading to EIDs include the acquisition of new pathogenicity and changes in the infection host. Nowadays, with climate change, increased interbreeding between vectors and hosts has greatly increased the likelihood of pathogen mutation [18]. Furthermore, globalization has led to increased trade and population movement between countries, facilitating the rapid propagation of mutant pathogens and increasing the possibility of an emerging pandemic [19].

COVID-19, which emerged at the end of 2019 and caused the most recent global pandemic, is a representative example of an EID. As of April 2025, approximately 777.7 million cases and 7.1 million deaths have been reported worldwide [20]. In the case of influenza, just a decade before the 2019 influenza pandemic, the world faced a serious health threat from a novel influenza pandemic. Furthermore, currently, there is a risk of another new influenza virus emerging through ongoing antigenic re-assortment and the movement of host cells [2,21,22]. Additionally, because of international trade and population movement between countries, reports of regional outbreaks of deadly hemorrhagic fevers, such as Ebola and Marburg, have occurred locally and have the potential to spread globally [3,23].

As we experienced during the COVID-19 pandemic, the emergence of novel infectious diseases creates gaps in disease management and healthcare due to the lack of diagnosis, prevention, and treatment strategies. This necessitates the development of proactive responses for global health security. To address this, the World Health Organization revealed a blueprint for research and development in the top 10 priority infectious diseases at the 68th World Health Assembly in Geneva, Switzerland, in 2015. These diseases include Crimean–Congo hemorrhagic fever, Ebola and Marburg viral diseases, Lassa fever, Middle East respiratory syndrome coronavirus (MERS-CoV) and severe acute respiratory syndrome coronavirus (SARS-CoV), Nipah and henipaviral diseases, Rift Valley fever, and disease X, a currently unknown pathogen with the potential to cause future epidemics (Table 1). This prioritized research is urgently required for global health security [24].

As observed in the case of COVID-19, the rapid development of vaccines is crucial for minimizing the damage caused by EIDs. In support of vaccine development for EIDs, the Coalition for Epidemic Preparedness Innovations (CEPI), a global partnership formed for this purpose, has set forth a 100-day mission to develop and distribute vaccines within 100 days to effectively respond to EIDs [32]. Consequently, the importance of faster and more efficient vaccine development technologies and platforms is being emphasized.

## 3. Vaccine Platforms for EIDs

**Figure 1:**
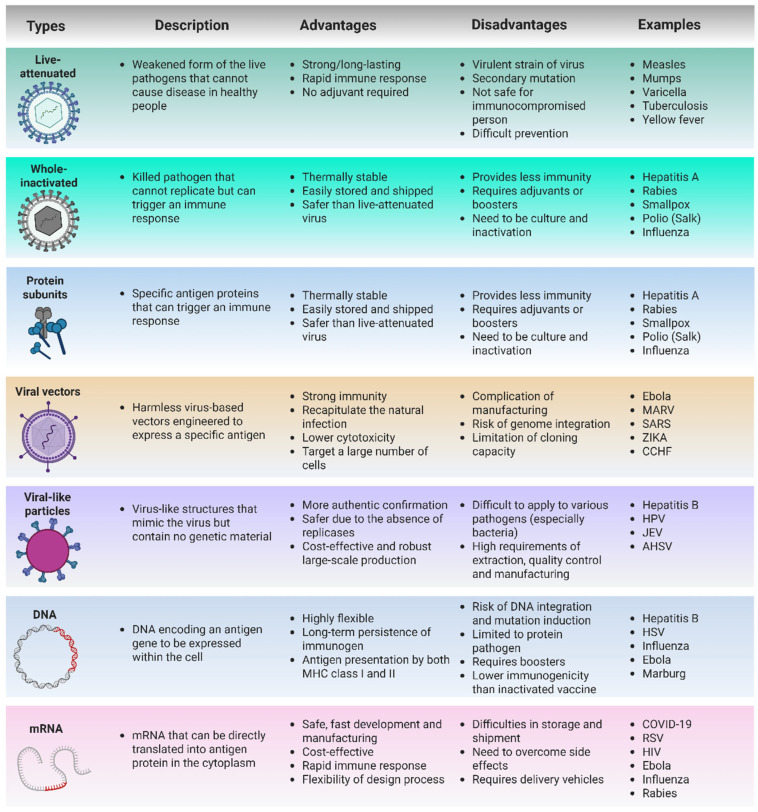
Advantages and disadvantages of various vaccine platforms for EIDs. Created with Biorender.com.

Vaccines stimulate and activate the immune system to combat infectious diseases, eradicate pathogens, and protect humans. Various vaccine development platforms exist, and they operate on the same fundamental principle. Through vaccination, the immune system recognizes pathogens (or a subunit of pathogens) and initiates an immune response against them. When the body is subsequently exposed to a similar pathogen, the immune system can induce a faster and stronger immune response to eliminate the pathogen. To date, many researchers have developed platforms for vaccine development, including whole-organism-based, live-attenuated, subunit, virus vector-based immunity, and nucleic acid-based (DNA and RNA) vaccines. We summarize the advantages and disadvantages of various vaccine platforms for EIDs in Figure 1.

### 3.1. Live-Attenuated and Whole-Inactivated Vaccines

The history of vaccine development began with the use of whole organisms that were live-attenuated and whole-inactivated. The methods involved acquiring attenuated organisms through continuous passage or inactivating pathogenic organisms via heat or chemical treatment to create whole-cell vaccines. Whole-cell vaccines were the initial vaccine approach for modern vaccination and are still used today to treat certain infectious diseases due to their relatively high efficacy and ability to induce long-lasting (in some cases, almost lifelong) immune responses [33,34,35,36]. The success of live-attenuated or whole-inactivated vaccines has significantly reduced the risk of infectious diseases, such as measles, mumps, varicella, smallpox, and polio, which posed serious health hazards in the premodern era.

In order to overcome the recent COVID-19 pandemic, various types of live-attenuated vaccines and inactivated vaccine formulations were developed, and some of them were used in various countries. Vaccines such as BBIBP-CorV (Sinopharm), CoronaVac (Sinovac), BBV152 (Bharat Biotech), and CoviVac (Chumakov Centre) were developed as inactivated virus formulations and were used in China, India, and Russia, respectively [37,38,39,40].

Although these have been useful in pandemic situations, whole-inactivated vaccines may require immune adjuvants or boosters because they may not elicit strong or long-lasting immunity compared with attenuated vaccines [41]. To overcome these limitations, Codagenix, Inc. developed COVI-VAC, a live vaccine attenuated through mutation, and it is conducting clinical trials for its use as a nasal vaccine [42]. However, live-attenuated vaccines can induce vaccine-derived infections caused by either the virus itself or secondary mutations that lead to virulence, making them unsafe for immunocompromised individuals [43]. Since most EIDs for which vaccine development is required are high risk, this safety shortcoming can act as a major obstacle to EID vaccine development. This highlights the need for safer vaccine formulations, leading to the development of other vaccine platforms.

### 3.2. Protein Subunit Vaccines

The primary function of a vaccine is to provide preemptive immunity against specific pathogen components. If we understand the critical aspects of the immune response, protective immunity can be generated by administering only relevant antigens (subunits) rather than the entire pathogen. Subunit vaccines, which do not involve the entire pathogen, offer protection against infectious diseases under safer conditions without the risk of whole-pathogen infection. Based on their safety and reliability, subunit vaccines, such as seasonal influenza and hepatitis B vaccines, are widely used [44,45]. However, unlike whole vaccines, an injection of the protein itself alone cannot induce an effective immune response, which is a key limitation of this type of vaccine that must be overcome. Therefore, this type of vaccine requires the use of an “immunoadjuvant” that can enhance and strengthen the immune response to the antigen [46,47]. Several immune adjuvants have been developed, building upon the initial approval of alum, with some showing successful effects [47,48].

During the COVID-19 pandemic, efforts were also made to develop vaccines using recombinant protein antigen formulations. NVX-CoV2373 (Novavax) and GBP510 (SK Biosciences) were developed with recombinant SARS-CoV-2 spike protein antigens mixed with Matrix-M and AS03 immune boosters, respectively, and they obtained clinical approval [49,50]. However, these vaccines have a lower market share than other vaccines due to their relatively late development. The development and production speed issues of recombinant protein vaccine formulations can be a disadvantage in responding to EIDs, where rapid development is important. In addition, immune adjuvants may not work effectively depending on the characteristics of the pathogens and the vaccine materials. Although the development of rapid protein production/purification technology and appropriate adjuvants can solve these problems, the application of a next-generation vaccine formulation that is rapid and has a higher immunogenicity could be another solution.

### 3.3. Viral Vector Vaccines

To overcome the low immunogenicity of subunit vaccines, various efforts have been made to apply viral vector formulations designed to express the subunit region of the target pathogen in other nonpathogenic viruses. It is known that viral vector-based vaccine formulations induce strong humoral and cellular immunity due to the characteristics of the vector itself [51]. Under this concept, modified vaccinia virus, adenovirus, Newcastle disease virus, and other viruses that do not cause symptoms in humans can be used as a vehicle for the expression of the target antigen of EIDs [52].

AZD1222 (AstraZeneca), which expresses the spike protein of COVID-19 using the chimpanzee adenovirus vaccine, played a critical role in the initial response to the COVID-19 pandemic [53,54]. In addition to COVID-19 vaccines, the development of viral vector-based vaccines targeting various other EIDs is ongoing. The Modified Vaccinia Ankara–Bavarian Nordic (MVA-BN) vaccine (Bavarian Nordic, Hørsholm, Denmark) is licensed as a prophylactic vaccine against smallpox and mpox (monkey pox) in the European Union (trade name IMVANEX^®^, Kvistgaard, Denmark) and the United States (trade name JYNNEOS^®^, Shreveport, LA, USA) [55]. Additionally, two types of Ebola hemorrhagic fever vaccines based on the viral vector platform have been developed and approved. rVSV-ZEBOV, developed by the Public Health Agency of Canada, is a modified vesicular stomatitis virus (VSV) that expresses the surface glycoprotein of Zaire Ebola virus and has been approved for use in the European Union and the United States [56,57]. The vaccine combination developed by Johnson & Johnson for Ebola prevention and approved in the EU consists of a human adenovirus serotype 26 vector formulation (Ad26.ZEBOV; Janssen Vaccines and Prevention, Dessau-Rosslau, Germany, as the prime) and a Modified Vaccinia virus Ankara vector formulation (MVA-BN-Filo; Bavarian Nordic, Hørsholm, Denmark, as a booster) [58]. Based on these results, efforts are underway to develop vaccines against other high-risk hemorrhagic fever viruses, such as Marburg and Crimean–Congo hemorrhagic fever (CCHF), using various viral vectors [59,60,61].

Despite these successful commercialization cases, viral vector-based vaccine formulations have several limitations. Viruses used as vectors can cause an excessive immune response, which can lead to side effects such as fever, pain at the injection site, and inflammatory reactions. In addition, viral vector vaccines have the characteristic of inducing immune responses induced by the viral vector itself rather than the target antigen, so the efficacy of the vaccine may be significantly reduced in cases of previous infection with the virus or repeated administration of the same viral vector formulation [62,63]. Therefore, another vaccine formulation that can overcome these limitations is required as an EID-responsive vaccine platform targeting a wide range of vaccine administration populations.

### 3.4. Virus-like Particle (VLP) Vaccines

The structural, envelope, or capsid proteins of a virus can spontaneously self-assemble to form virus-like particles (VLPs) without the viral genome. The virus-like particles/viral-like particles (VLPs) formed in this way mimic the overall structure of virus particles but do not contain infectious genetic material or virulence factors. Because of these properties, VLPs have garnered attention as attractive vaccine formulations because they can induce immune responses similar to actual infection situations without the risk of infection [64]. Preventive vaccines against hepatitis B virus (HBV), hepatitis E virus (HEV), and human papillomaviruses (HPVs) have been developed in VLP formulations and are currently in use [65].

In the development of EID vaccines, several clinical/pre-clinical studies examining candidates based on VLP formulations are currently underway. VLP formulations composed of SARS-CoV-2 and chikungunya virus envelope proteins have already entered clinical trials [66,67], and VLP-based vaccine candidates targeting Zika virus, Dengue virus, Ebola virus, and Nipah virus are reporting promising results in nonclinical studies [68,69,70,71,72].

However, VLP vaccines have the disadvantage that they are difficult to apply to bacterial infections. Bacteria are much larger and more complex in structure than viruses, making it difficult to mimic the complexity of bacteria with VLPs. In addition, bacterial antigens are diverse and often have excellent immune evasion mechanisms, making it difficult to select and express appropriate antigens. The fact that bacteria often use toxins as their main pathogenic factors also makes it difficult for the simple external mimicry of VLPs to provide a sufficient immune defense. It is not that there have been no efforts to develop VLP vaccines against bacterial infections, but rather that the formulation characteristics of VLP vaccines, which only mimic the external structure of pathogens, act as a major limiting factor in the development of bacterial vaccines.

In addition, VLP production requires a lot of time and investment. Since much preliminary research is required to establish the production conditions of VLPs to induce complete immunogenicity, there are limitations in using them for the development of EID vaccines, where rapid development and production are important.

### 3.5. DNA Vaccines

DNA formulations, which emerged as potential vaccines in the 1990s, became an attractive vaccine platform for EIDs because of their scalability, simplicity, and stability. DNA vaccines can be rapidly designed and produced, adapting promptly to EIDs with known and concerning genetic sequences [20,73]. With the development of methods to increase gene expression and immunogenicity, including codon and plasmid backbone optimization [74], recombinant DNA constructs involving promoter replacement [75,76], and the co-encoding of genetic adjuvants such as pattern recognition receptors (PRRs) and cytokines [77,78], DNA vaccine formulations have emerged as a potential new vaccine platform for combating EIDs.

For HIV (human immunodeficiency virus), which still has no preventive vaccine despite extensive research, the clinical applicability of DNA vaccine candidates (DNA-HIV-PT123) is currently being investigated [79]. In the case of the Zika virus, which was designated as a public health emergency of international concern by the WHO in 2016, several candidates based on DNA formulations have entered clinical trials as preventive vaccines (ClinicalTrials.gov registration numbers: NCT02809443, NCT02840487, and NCT02996461) [80,81]. In addition, several vaccine candidates for various EIDs, such as West Nile virus, Hantann virus (HTNV), and Puumala virus (PUUV), have been developed as DNA vaccine formulations and have entered the clinical trial stage [82,83].

However, the current DNA vaccine formulation has several challenges to overcome. First, DNA vaccines are still considered to have relatively low immunogenicity compared to other vaccine platforms, and this is considered to be due to limitations in the delivery technology of DNA formulations [84]. Due to the nature of DNA vaccines, which must be delivered to the nucleus to induce antigen expression, it is difficult to expect antigen expression on a sufficient scale through the administration of naked DNA. To solve this problem, the applicability of various injection/stimulation methods, such as gene guns, tattooing, microneedling, electroporation, sonoporation, and photoporation, in addition to traditional needle injection, is being studied [85], but much additional research is still needed for new technologies to be applied in actual clinical practice. Another hurdle facing DNA vaccines is safety issues. Although rare, the possibility that DNA injected into the body may integrate into the host cell genome carries a serious risk of mutation [84]. This is an innate limitation of this formulation that has been continuously pointed out since the development of DNA vaccines and is an element that must be overcome in order to develop a safe vaccine.

### 3.6. mRNA Vaccines

#### 3.6.1. Characteristics of mRNA Vaccine Formulations

mRNA has traditionally been studied for cancer immunotherapy or to supplement defective proteins, and the potential of mRNA formulations for use as vaccines was first noted in the 1990s. Research on the formulation characteristics of mRNA, accumulated through numerous related studies, has led to the emergence of mRNA vaccines, which played a pivotal role in the COVID-19 pandemic [86,87]. mRNA vaccines are composed of single-stranded RNA encoding the antigen of interest, which is generally encapsulated in vehicle materials, such as lipid nanoparticles (LNPs), to facilitate its delivery and internalization into cells. Once delivered into the cytoplasm, the mRNA is directly translated into protein by the host cell’s natural translational machinery, undergoing post-translational modification to achieve full functionality. In addition, the mRNAs administered through vaccines can be recognized as exogenous RNAs in host cells, inducing strong immunostimulation without other adjuvants [88]. In particular, RNA-induced immunostimulation is known to effectively induce Th1-biased immune responses [89]; thus, it can be effectively applied to infectious disease vaccines that require the induction of a cellular immune response.

Similar to DNA vaccines, mRNA vaccines have the advantages of scalability and simplicity related to the nucleic acid vaccine formulation. However, in contrast to DNA vaccines, mRNA vaccines have no risk of host genome mutations because of the transient nature of mRNA, and they do not induce a prolonged expression of foreign genes, resulting in relatively fewer side effects. In addition, mRNA can be translated directly in the cytoplasm and express antigens without needing to be delivered to the nucleus, so a higher immunogenicity can be expected compared to DNA vaccines [90]. For these reasons, currently, many vaccines, such as those for universal influenza, tuberculosis, and high-risk pathogens, are being developed based on mRNA formulations [91,92,93,94,95,96].

mRNA vaccines are also considered the most suitable vaccine type for the response to EIDs. mRNA vaccines offer significant advantages over recombinant protein or viral vector vaccines as a response strategy for EIDs, which require rapid manufacturing and production. mRNA vaccines also have the flexibility to respond quickly to variant strains via the simple substitution of the target open reading frame (ORF) region. Considering that EIDs pose a more severe threat in low- and middle-income countries where access to medical systems is difficult, the relatively low production costs of mRNA vaccine formulations make them ideal as public vaccines. Based on the various strengths of mRNA vaccine formulations over conventional vaccine formulations (Table 2), mRNA vaccines have emerged as the most promising formulation since the COVID-19 pandemic

However, current mRNA vaccines require cold chains for storage and distribution because of their low stability and susceptibility to degradation. In addition, although many people were vaccinated with mRNA vaccines manufactured by Pfizer/BioNTech and Moderna during the COVID-19 pandemic, clinical data on their efficacy and safety are still lacking because this new formulation has only recently begun to be used. Furthermore, because vaccine approval and commercialization are first-time events, established systems, standards, and methods to verify vaccine efficacy and safety are lacking. Therefore, thoroughly understanding the ingredients and characteristics of mRNA vaccine preparations and establishing appropriate analysis techniques suited to these characteristics are urgent tasks.

#### 3.6.2. Modified mRNA Vaccines

Traditional mRNA vaccines are generally designed and formulated to mimic the function of mRNA within the body, possessing a structure identical to that of intracellular mRNA. These vaccines are referred to as nonreplicating mRNA (nrRNA) vaccines, as they immediately synthesize protein upon introduction into the body without undergoing a replication process [90]. Vaccines with nrRNA formulations currently constitute the majority of mRNA vaccine types in use. However, there remains a need to enhance the stability and immunogenicity of these formulations to achieve superior vaccine efficacy.

One of the most important factors that has enabled the clinical use of mRNA formulations with very easily degradable properties has been the development of carriers that safely protect and deliver nucleic acids [97]. LNPs, which are currently used as the main carrier, have a spherical nanoscale structure composed of ionizable cationic lipids, cholesterol, phospholipids, and PEG–lipids. This carrier effectively protects mRNA from degradation by nucleases while promoting efficient cellular uptake and the cytoplasmic release of mRNA [98]. However, many of the side effects reported after mRNA vaccine administration, such as inflammation, hepatotoxicity, and anaphylactic reactions, are considered to be caused by LNPs [99]. In addition, the mRNA-LNP complex is thermodynamically unstable [100], so ultra-low temperature conditions are essential for distribution, which acts as a major barrier to expanding vaccine accessibility and reducing costs. To solve these problems, various studies are actively being conducted to incorporate new types of carriers, such as polymeric nanoparticles, polysaccharide-based nanoparticles, carbon-based nanoparticles, dendrimers, metal-based nanoparticles, liposomes, and extracellular vesicles, into mRNA vaccines [101].

In addition to modifying the carrier materials, efforts are also actively being made to improve mRNA vaccine formulations through modification of the nucleic acid itself. Immunogenicity can be enhanced by increasing the quantity of the administered antigen; however, any increase in vaccine material dosage must be carefully considered due to the risk of side effects from an increase in co-administered auxiliary materials. Self-amplifying RNA (saRNA) formulations aim to minimize such risks by expressing an enzyme involved in self-amplification (e.g., the RNA replicase of alphavirus), enabling the administered mRNA to increase antigenicity through multiple rounds of replication without dose escalation [102,103]. Recently, an saRNA vaccine developed by HDT Bio for COVID-19 prevention has been approved and launched [104], and other saRNA-based vaccine products, such as Pfizer’s seasonal influenza vaccine (PF-07845104), are also being examined in clinical trials [105]. However, in the case of saRNA, additional ORFs coding for replication-related enzymes and target proteins need to be included in the genome, leading to an increase in genome size. As a result, challenges arise in the encapsulation of LNPs, secondary structure formation, and decreased translational efficiency. To address these issues, additional studies are currently underway on the efficiency of trans-amplifying RNA (taRNA), which separates the genome expressing the replication-related ORF from the target protein [106,107].

Traditional mRNA vaccine formulations have a linear structure, rendering them susceptible to degradation by nucleases present in the body. To mitigate these drawbacks, active research is being conducted to produce a circular form (cirRNA) to bolster the stability of intracellular RNA [108,109]. Although cirRNAs are still in the early stages of development, they are viewed as a highly promising technology. Moreover, cirRNAs are relatively unencumbered by patent disputes, a significant hurdle in the development of conventional mRNA formulation vaccines, leading numerous pharmaceutical companies to invest heavily in cirRNA vaccine development. The advantages and disadvantages of each mRNA vaccine formulation are summarized in Table 2.

#### 3.6.3. Current Status of mRNA Vaccine Development for Various EIDs

The successful development of mRNA vaccines by Pfizer/BioNTech and Moderna in response to the COVID-19 pandemic has been a groundbreaking achievement, sparking research into the development of vaccines for various infectious diseases based on mRNA formulations. Several mRNA vaccines targeting SARS-CoV-2 and its variants have already received approval and have been commercialized, with numerous clinical trials in progress for mRNA vaccines targeting other infectious diseases. Although various companies across several countries are developing mRNA vaccines for COVID-19 prevention, to date, mRNA vaccines targeting infectious diseases other than COVID-19 are primarily spearheaded by leading companies such as Moderna, Pfizer/BioNTech, and CureVac. Currently, a number of mRNA vaccines targeting EIDs (COVID-19, pandemic flu, Lyme disease, Zika, Nipah, chikungunya, etc.) are entering the clinical stage. Table 3 provides an overview of the infectious diseases (including EIDs) targeted by mRNA vaccines currently being examined in clinical research by major companies.

## 4. Conclusions and Perspective

Today, the emergence of EIDs is becoming increasingly frequent, with some of them causing global pandemics. As a result, humanity is currently facing a serious health threat, and the preparation of countermeasures to resolve this is urgently required. As seen in the recent process of overcoming the COVID-19 pandemic, vaccination during the early stages of a pandemic is the most effective and essential strategy to prevent the spread of EIDs. To date, various vaccine formulations have been developed, laying the foundation for a preemptive response to EIDs. Among them, mRNA vaccine formulations offer significant advantages, such as rapid development and production, over other vaccine platforms. They played a pivotal role in curbing the spread of infectious diseases during the global COVID-19 pandemic. mRNA vaccines are considered the optimal vaccine platform for EIDs, where a rapid response is paramount. As the scope of applications of mRNA vaccine formulations expands, they are expected to be the focus of the response to future EIDs.

However, the clinical use of mRNA vaccines is just in its beginning stages, and systems for evaluating vaccines based on mRNA formulations are still lacking, thus presenting a major obstacle to the rapid application of mRNA vaccines to EIDs. In order to continue to use mRNA vaccine preparations as a future EID response strategy, it is necessary to develop an analysis system that can verify the effectiveness and safety of the mRNA vaccine, as well as the development process of the vaccine itself. At the same time, it is important to consider standardizing and streamlining the clinical trial and approval process based on the formulation characteristics of the mRNA vaccine.

## Figures and Tables

**Table 1:** The list of priority diseases from the WHO blueprint for R&D, modified from [25].

Disease	Fatality Rate (%)	Recent Outbreak	References
CCHF	>30	Uganda, 2018–2019	[4]
Ebola viral disease	32–90	Uganda and Democratic Republic of Congo, 2022	[26]
Marburg viral disease	23–90	Tanzania, 2023	[27]
Lassa fever	~1 (infected cases) ~20 (hospitalized cases)	Annually recurring outbreaks in West Africa	[28]
MERS	~35	Saudi Arabia, 2013–2018 South Korea, 2015	[25]
SARS	~10	Global, 2003	[25]
Nipah and henipaviral diseases	~30	India, 2018	[29]
RVF	<1	Uganda, 2022–2023	[30]
Zika disease	Not fatal	India, 2021	[31]
Disease X			[25]

CCHF, Crimean–Congo hemorrhagic fever; MERS, Middle East respiratory syndrome; SARS, severe acute respiratory syndrome; RVF, Rift Valley fever.

**Table 2:** Advantages and disadvantages of types of mRNA vaccines for EIDs.

Type of mRNA Vaccine	Description	Advantages	Disadvantages
Non-replicating mRNA (nrRNA)	Basic formulation of mRNA vaccine	Relatively simple structure	Requires a high dose for induction of sufficient immune response and vulnerable to degradation by RNase
Self-amplifying RNA (saRNA)	Expresses enzymes involved in self-amplification	High antigen expression with low-dose vaccination	Increases the size of the mRNA due to the ORF for replication, resulting in reduced encapsulation and translation efficiency
Trans-amplifying RNA (taRNA)	Self-replicating mRNA in which the replication ORF and antigen ORF are separated	Reduction in individual mRNA size through separation of ORF for antigen and replication enzyme expression	The complexity of mRNA design and the need to confirm the proper content ratio of individual RNAs
Circular mRNA (cirRNA)	Circular nucleic acids for enhanced intracellular stability	Relatively stable from degradation by RNase	Requires additional research on expression and encapsulation efficacy

**Table 3:** Status of mRNA vaccine development for infectious diseases by major pharmaceutical companies (infectious diseases in bold are considered EIDs) ^a^.

Developer	Target Infectious Disease	Vaccine Name (Temporary Name)	Development Stage	Clinicaltrials.Gov ID (in Case of Clinical Trials in Progress)
Moderna (Cambridge, MA, USA)	**COVID-19** (adults/adolescents)	Spikevax^®^	Registration	-
	**COVID-19** (pediatrics)	mRNA-1273.815	Registration	
	**COVID-19** (adults)	mRNA-1283	Phase 3	NCT05815498
	**COVID-19** (adolescents)	mRNA-1273/mRNA-1273.214/.222	Registration	-
	**COVID-19** (pediatrics)	mRNA-1273/mRNA-1273.214/.222	Registration	-
	Influenza	mRNA-1010	Phase 3	NCT05827978
	Influenza	mRNA-1020	Phase 1/2	NCT05333289
	Influenza	mRNA-1030	Phase 1/2	
	Influenza	mRNA-1011	Phase 1/2	NCT05827068
	Influenza	mRNA-1012	Phase 1/2	
	RSV (older adults)	mRESVIA^®^	Registration	
	RSV	mRNA-1345	Phase 3	NCT06067230
	RSV + HMPV	mRNA-1365	Phase 1 (Child)	NCT05743881
	Flu + **COVID-19**	mRNA-1083	Phase 3	NCT06694389
	Flu + **COVID-19** + RSV	mRNA-1230	Phase 1 (Adult, Older Adult)	NCT05585632
	Flu + RSV	mRNA-1045	Phase 1	NCT05585632 NCT05972174
	**Pandemic flu**	mRNA-1018	Phase 1/2	
	CMV	mRNA-1647	Phase 3	NCT05085366
	EBV (prevent infectious mononucleosis)	mRNA-1189	Phase 1	NCT05164094
	EBV (prevent long-term EBV sequelae)	mRNA-1195	Phase 1	NCT05831111
	HSV-2	mRNA-1608	Phase 1/2	NCT06033261
	VZV	mRNA-1468	Phase 1/2	NCT05701800
	HIV	mRNA-1644	Phase 1	NCT05414786, NCT05001373
	HIV	mRNA-1574	Phase 1/2	NCT03619278
	Norovirus	mRNA-1403	Phase 3	NCT06592794
	Norovirus	mRNA-1405	Phase 2	NCT05992935
	**Lyme disease**	mRNA-1975	Phase 1/2	NCT05975099
	**Lyme disease**	mRNA-1982	Phase 1/2	NCT05975099 NCT04917861
	**Zika**	mRNA-1893	Phase 2	
	**Nipah**	mRNA-1215	Phase 1	NCT05398796
	**Mpox**	mRNA-1982	Phase 1/2	NCT05975099
	**Chikungunya**	mRNA-1944	Phase 1	NCT03829384
	**Chikungunya**	VAL-181388	Phase 1	NCT03325075
	**COVID-19**	COMIRNATY^®^ (BNT162b2)	Registration	-
Pfizer (Manhattan, NY, USA) and/or BioNTech (Mainz, Germany)	**COVID-19**	BNT162b2 bivalent (BA.4/BA.5)	Registration	-
	**COVID-19**	BNT162b2 bivalent (BA.4/BA.5)	Registration	-
	**COVID-19**	Omicron XBB.1.5-Adapted Monovalent	Registration	-
	Influenza	PF-07252220	Phase 3	NCT05540522
	Influenza	9 kinds of influenza saRNA	Phase 1	NCT05227001
	**COVID-19** + flu	BNT162b2 + BNT161	Phase 1/2	NCT05596734
	Varicella	PF-07911145 (BNT167)	Phase 1/2	NCT05703607
	HSV-2	BNT163	Phase 1	NCT05432583
	Tuberculosis	BNT164a1, BNT164b1	Phase 1/2	NCT05537038, NCT05547464
	HPV	BNT113	Phase 2	NCT04534205
	Malaria	BNT165b1	Phase 1/2	NCT05581641
	**Mpox**	BNT166	Phase 1/2	NCT05988203
	Shingles	BNT167	Phase 2	NCT05703607
	Influenza	CVSQIV	Phase 1	NCT05252338
CureVac (Tuebingen Germany) and/or Glaxo SmithKline (London, UK)	**COVID-19**	CV0501	Phase 1	NCT05477186
	**COVID-19**	CV2CoV (GSK4396687)	Phase 1	NCT05260437
	Rabies	CV7202	Phase 1	NCT03713086
	**Avian influenza**	GSK4382276	Phase 1/2	NCT05823974

^a^ Data from the homepage of each company and the data source: https://clinicaltrials.gov/ (accessed on 1 April 2025). COVID-19, coronavirus disease 2019; RSV, respiratory syncytial virus; CMV, cytomegalovirus; EBV, Epstein–Barr virus; HSV-2, herpes simplex virus-2; VZV, varicella zoster virus; HIV, human immunodeficiency virus; HPV, human papillomavirus; HMPV, human metapneumovirus.
